# Translation-complex profiling of fission yeast cells reveals dynamic rearrangements of scanning ribosomal subunits upon nutritional stress

**DOI:** 10.1093/nar/gkac1140

**Published:** 2022-12-08

**Authors:** Caia Deborah Suzanne Duncan, Juan Mata

**Affiliations:** Department of Biochemistry, University of Cambridge, Cambridge CB2 1QW, UK; Department of Biochemistry, University of Cambridge, Cambridge CB2 1QW, UK

## Abstract

Control of mRNA translation is key for stress responses. Translation initiation is usually rate-limiting and, in eukaryotes, involves mRNA scanning by the small ribosomal subunit. Despite its importance, many aspects of translation *in vivo* have not been explored fully, especially at the transcriptome-wide level. A recent method termed translation-complex profiling (TCP-seq) allows transcriptome-wide views of scanning ribosomal subunits. We applied TCP-seq to nutritional stress in the fission yeast *Schizosaccharomyces pombe*. At initiation sites, we observed multiple complexes resembling those of mammals, and consistent with queuing of scanning subunits. In 5′ UTRs, small subunit accumulations were common and may reflect impediments to scanning. A key mediator of stress responses in *S. pombe* is the Fil1 transcription factor, which is regulated translationally by a poorly-understood mechanism involving upstream Open Reading Frames (uORFs). TCP-seq data of *fil1* shows that stress allows scanning subunits to by-pass specific uORFs and reach the *fil1* coding sequence. The integration of these observations with reporter assays revealed that *fil1* translational control is mediated by a combination of scanning reinitiation-repressive and permissive uORFs, and establishes *fil1* as a model for uORF-mediated translational control. Altogether, our transcriptome-wide study reveals general and gene-specific features of translation in a model eukaryote.

## INTRODUCTION

A rapidly-growing bacterial cell devotes 50% of its energy consumption to mRNA translation (1), and a differentiating mammalian cell 30%(2). Consistent with this major energy expense, regulation of translation is key for cell survival both at the global level (total output) and for individual genes. This is particularly important under situations of stress (3,4), where resources devoted to translation may need to be reallocated.

Translation in eukaryotes starts with the formation of a 43S preinitiation complex, consisting of the small ribosomal subunit (40S) and the initiation factors (eukaryotic initiation factors or eIFs) eIF3, eIF1, eIF1A and the ternary complex eIF2–GTP–Met–tRNA_*i*_^Met^ (TC)(5). The 43S complex then binds to eIF4G on the cap, leading to the formation of the 48S complex, which scans the mRNA until it reaches the first initiation codon in a good sequence context. A number of rearrangements then lead to the release of eIFs and the recruitment of the 60S subunit (6,7).

Eukaryotic mRNAs often contain short open reading frames in their leader sequences (upstream open reading frames, or uORFs). Translation of uORFs in yeasts is often inhibitory, as it may prevent the scanning complexes from reaching the major coding sequences (8). However, uORF-dependent blocks can be by-passed, either by leaky scanning (in which the uORF start codon is not used) or by reinitiation (in which scanning resumes following translation of the uORF). The ability to reinitiate scanning is dependent on *cis* elements (mRNA features flanking or within the uORF (9)), the length of the uORF, the distance between the previous translation stop and the uORF, and the actions of trans factors (some eIFs that remain associated with translating ribosomes in early elongation (10–12)). In addition, scanning reinitiation requires the reacquisition of eIFs that dissociate during the translation of the uORF, including the TC (8).

uORFs can be used to regulate translation in response to stress. The best understood system of uORF-mediated regulation of translation is that of the *GCN4* gene from *Saccharomyces cerevisiae* (13). The *GCN4* leader sequence contains four uORFs, two of which behave as repressive for reinitiation and two that behave as permissive. However, a simplified system consisting of uORF1 (permissive, i.e. that promotes reinitiation) and uORF4 (repressive, i.e. that promotes termination) can recapitulate the endogenous regulation (although the complete system may be more robust)(14). Reinitiating complexes formed after uORF1 translation need to reacquire an active TC (i.e. GTP-bound) before they can translate uORF4 or the main ORF. Under non-stressed conditions, active TC is abundant and is recruited to the reinitiating complex in time to translate repressive uORF4, thus preventing *GCN4* translation. In stress conditions, low levels of active TC prevent translation of uORF4, and allow the translation of *GCN4* by the reinitiating complexes that bypass uORF4 and acquire the TC before reaching the main ORF. The abundance of active TC is regulated by a conserved signalling pathway called the Integrated Stress Response (ISR), under the control of eIF2α kinases (GCN2 in *S. cerevisiae*) (15). A similar pathway is present in mammals and regulates the expression of the transcription factor ATF4, although the details of the mechanism differ from those of *S. cerevisiae (*16,17). uORF-mediated regulation of translation has been studied in detail for only a few eukaryotic mRNAs.

The fission yeast *S. pombe* has been used as an alternative and complementary model to the study of translational control in response to stress (18–26). The composition of the eIF3 complex of *S. pombe* and mammals is similar, containing both core (a, b, c, e, h) and accessory subunits (13 in mammals and 10 in *Schizosaccharomyces pombe*), whereas the *S. cerevisiae* complex is limited to the 5 core components (27). Another example is the programmed frameshifting in the production of ornithine decarboxylase antizyme, which is mostly + 1 in *S. pombe* and mammals, but -2 in *S. cerevisiae* (28). Finally, the *S. pombe* gene *fil1* encodes a transcription factor whose translation is up-regulated in response to multiple stresses, most notably amino acid starvation where up-regulation is dependent on the Gcn2 kinase (21,24). Here translational control is mediated by up to 6 uORFs in its 5′ UTR, although the precise mechanism remains unknown. Although Fil1 is not homologous to mammalian ATF4 or *S. cerevisiae* GCN4, it behaves as a functional ortholog (21) whose major function is the activation of the transcription of amino acid biosynthesis genes. Another interesting case of the convergent use of uORFs for translation regulation under stress is the expression of *S*-adenosylmethionine decarboxylasein response to polyamines, which is mediated by uORFs in both plants (29) and mammals (30) despite the lack of a common origin.

The invention of ribosome profiling has provided unprecedented views of translation at the transcriptome level (31). The approach is based on the treatment of polysome-containing cell extracts with a ribonuclease (RNase I). RNA fragments protected by ribosomes (footprints or FPs) survive the RNase treatment and are isolated and identified by high throughput sequencing. The location of the FPs provides information of the location of the ribosome on mRNAs with single-nucleotide resolution. However, as some scanning ribosomal subunits are released from mRNAs during the process, they are not amenable to investigation by ribosome profiling (32). This limitation was addressed by a novel approach called Translation Complex Profiling analysed by sequencing (TCP-seq) (32). In this method, translation complexes are stabilised by cross-linking *in vivo*. Cell extracts are treated with RNase and 40S and 80S ribosomes are separated using sucrose gradients (7). TCP-seq or related methods have only been applied to a few organisms and conditions (10,11,33,34).

We have optimised the TCP-seq protocol for *S. pombe* and applied it to the study of amino acid starvation. Our results reveal features of translation complexes, both global and for individual genes. Moreover, the combination of TCP-seq and reporter assays uncover the regulation on the uORF-mediated regulation of the *fil1* gene.

## MATERIALS AND METHODS

### Modified TCP-seq protocol

100 mL of 30% paraformaldehyde (PFA) per sample was freshly prepared as described (35). A final concentration of 10 mM 3-amino-1,2,4-triazole (3-AT) was added to cell cultures 1 h prior to harvest. Cells were cultured in 0.8 l EMM2 media at 32°C to an OD between 0.45–0.8 then snap cooled by adding 250 g crushed ice in a cold beaker with constant stirring. 100 ml of 30% PFA was added and quenched after 10 min with the addition of 60 ml 2.5 M glycine. Cells were pelleted for 2 min at 2000g at 4°C and washed twice in buffer containing 25 mM HEPES–KOH pH 7.6, 100 mM KCl, 2 mM MgCl_2_ and 250 mM glycine, aliquoted into 10 vials and frozen on dry ice. For isolation of 40S and 80S complexes, 4–5 vials of cells were lysed in 25 mM HEPES–KOH pH 7.6, 100 mM KCl, 2 mM MgCl_2,_ 0.25% Triton-X100, 0.5 mM DTT, 250 mM glycine, Complete Mini EDTA Free Protease Inhibitor Cocktail (Roche), 10 mM PMSF, 100 μg/ml cycloheximide, 100 U RiboLock RNase inhibitor (Thermo Fisher Scientific), and 1 U TURBO DNase (Thermo Fisher Scientific). Each vial of cells was lysed with 100 μl of lysis buffer and 1 ml of acid washed 0.5 mm glass beads in a FastPrep-24 5G (MP Biomedicals) at level 7 for 13 s. 200 μl of lysis buffer was added before samples were pooled and cleared at 12 500 rpm for 5 min and then 10 min at 4°C. Samples were digested with 550 U Ambion RNase I (Thermo Fisher Scientific) for every 1000 *A*_260_ units of lysate for 45 minutes at 23°C. Up to 1200 *A*_260_ units were loaded onto each gradient. Sucrose gradients were prepared using the SW40 adaptor on the BioComp Gradient Master. 7.5 and 30% sucrose solutions were made in 50 mM Tris–HCl pH 7.0, 50 mM NH_4_Cl, 4 mM MgCl_2_, 100 μg/ml cycloheximide and 0.5 mM DTT. Samples were separated in an SW40Ti rotor at 37 000 rcf for 4 h at 4°C and fractionated on a Brandel density Gradient fractionator into 400 μl fractions. Fractions containing the 40S or 80S complex were pooled and incubated with an equal amount of acid phenol:chloroform:isoamyalcohol pH 4.5 (125:24:1), 1% SDS and 125 mM glycine at 65°C for 45 min. The aqueous layer was then cleaned with room temperature phenol: chloroform pH 4.5 followed by chloroform before isopropanol precipitation with GlycoBlue (Thermo Fisher Scientific). RNA fragments between 15–100 nucleotides were purified from the 40S and 80S RNAs on 10% TBE–urea gels (Thermo Fisher Scientific). RNAs were treated with T4 polynucleotide kinase (PNK, Thermo Fisher Scientific) at pH 7.0 for 30 min followed by 30 min at pH 7.6 with 10 mM ATP. NGS libraries were made with the NEXTFlex Small RNA-seq kit v3 following the no size selection protocol using up to 100 ng of starting material. 8–10 cycle PCR products were quantified using the Qubit dsDNA high sensitivity kit (Thermo Fisher Scientific) and the ProNex® NGS Library Quant Kit (Promega). Illumina NovaSeq 6000 sequencing was carried out by the CRUK-Cambridge Institute Genomics Core.

Total RNA was extracted from 1 vial of cross-linked cells resuspended in 10 mM Tris pH 7.5, 10 mM EDTA, 0.5% SDS and RNA was extracted with hot acid phenol:chloroform:isoamylalcohol pH 4.5 (125:24:1). 1 μg of RNA was treated with 4 U of TURBO DNase (Thermo Fisher Scientific) for 1 h at 37°C, followed by RQ1 DNase (Promega) for 1 h at 37°C. RNAs were purified using PureLink RNA microcolumns (with on-column DNase treatment, Thermo Fisher Scientific). Samples were rRNA-depleted using NEBNext® RNA Depletion Core Reagent Set (NEB) with yeast probes. NGS libraries were made using NEXTFLEX™ Rapid Directional qRNA-Seq™ Kit v2 (BIOO Scientific) with 30 ng of depleted RNA as the starting material. Twelve cycle PCR products were quantified using the Qubit dsDNA high sensitivity kit (Thermo Fisher Scientific) and the ProNex® NGS Library Quant Kit (Promega). Illumina NovaSeq 6000 sequencing was carried out by the CRUK-Cambridge Institute Genomics Core.

### Reporter construction and flow cytometry

The reporter constructs were cloned using the pDUAL plasmid back bone (36) and contained the *adh1* promoter sequence and relevant *fil1* 5′ UTR sequence upstream of a ubiquitin—heat degron (37) (DHFR)—VenusNB coding frame (38). The *fil1* 3′UTR and terminator sequences were inserted after the coding frame. All cloning was performed with NEBuilder HiFi DNA Assembly master mix (NEB). uORFs AUG (CUG for uORF1) sequences were mutated to AAA with overlapping primers. Plasmids were digested with NotI-FD (Thermo Fisher Scientific) and transformed into *h- leu1-32* for integration into the *leu1* locus. Reporter strains were grown in EMM2, diluted to OD 0.2, grown for 1 h and treated with 3-AT to a final concentration of 10 mM. After 5 h, 10 ml cells were pelleted and frozen for RNA extraction, while 300 μl were fixed with cold ethanol to a final concentration of 70%. Fixed cells were hydrated for more than 1 h in PBS before analysis on the CytoFlex S Flow cytometer (Beckman Coulter). **RT-qPCR**: Cell pellets were resuspended in 10 mM Tris pH 7.5, 10 mM EDTA, 0.5% SDS and RNA was extracted with hot acid phenol:chloroform:isoamylalcohol pH 4.5 (125:24:1). Samples were incubated with 2 U TURBO DNase (Thermo Fisher Scientific) for 1 h at 37°C, purified on PureLink RNA mini columns (with additional on-column DNase step, Thermo Fisher Scientific). Purified RNA was measured by nanodrop and 2 μg of each RNA was incubated at 37°C with 1 U RQ1 RNase-free DNase (Promega) for 1 h. 100 ng of DNase-treated RNA was reverse-transcribed with random hexamers using superscript II (Thermo Fisher Scientific) following the manufacturer's instructions and diluted 1 in 20 with water. qPCR was performed in triplicate using qPCRBIO SyGreen Mix (PCR BioSystems) and primers to the Venus coding sequence and to the endogenous *adh1* coding sequence as a reference gene, as the reporter construct utilizes the *adh1* promoter sequence. PCR efficiency for Venus was 0.9546 (Venus_F: GGTGATACTCTTGTCAACCGC, Venus_R: CAGCAAGTTGAACACCACCA, amplicon size: 193 nt) and PCR efficiency for *adh1* is 1.004 (*adh1*_F: GATGCCTTTGATCGGTGGTC, *adh1*_R: TGAATGTGAGGGCAGATGGT, amplicon size: 168 nt). dCq values were first calculated between Venus and *adh1* within each sample. ddCq was then calculated for the response to 3-AT. Three independent biological replicates were performed.

### Analysis methods

Processing of RNA libraries was performed with Perl custom scripts. Illumina RNA-seq libraries were sequenced as paired-end with a length of 75 bp (PE75). The structure of the forward reads was as follows: RRRRRRRRA(NNNN….NNNN), where R (8) corresponds to a random sequence from a set of 256, A to an adenosine residue, and N to the sequence of the RNA fragment. The structure of the reverse reads was similar except that the adenosine is replaced with a thymidine. The random sequences of both reads were merged to create single unique molecular identifiers (UMI), which was used to identify and remove duplicated reads. Processed sequences were mapped to the *S. pombe* transcriptome (39) using STAR version 2.7.3a (40) with –outFilterMismatchNmax 5 and otherwise default parameters. Illumina TCP-seq libraries were sequenced as single-end 100 nucleotides (SE100). The structure of the reads was as follows: RRRR(NNNN…NNNN)RRRR-adaptor-, where R represents random nucleotides; N corresponds to the sequence of the RNA protected fragment; and the Illumina adaptor sequence is TGGAATTCTCGGGTGCCAAGG. The random nucleotides served as unique molecular identifiers (UMIs) and used to remove PCR duplicates. Adaptor sequences were removed with Cutadapt (41). Processed sequences were mapped to the *S. pombe* rRNA genome using STAR version 2.7.3a (40) with –outSAMmultNmax 1, –outFilterMultimapNmax 50 and otherwise default parameters. Unmapped reads were mapped to the *S. pombe* transcriptome with—outFilterMismatchNmax. Transcriptome files for mapping were generated by merging three fasta files downloaded from PomBase (09/05/2021) (39) containing 5′ UTRs, 3′ UTRs and coding sequences. One hundred base pairs were added to the 5′ end of all annotated 5′ UTRs. CAGE data were from Li et al (42). Libraries from TCP-seq experiments tend to be small. To increase the quality and representativity of the dataset, we prepared multiple libraries from some biological samples. Libraries from the same sample were processed separately and merged at the post-alignment stage (i.e. as bam files). Identification of differentially expressed genes was performed with the RNA-seq dataset and DESeq2(43), using an adjusted *P*-value of 0.01 and a minimal 2-fold change. For differentially translated genes ΔTE (44) was used with an adjusted *P*-value of 0.025 and no fold-change threshold. Only reads from the 80S libraries that mapped to coding sequences were considered. For consistency, unless otherwise indicated, all heat maps shown are from replicate number 2, untreated cells. Similar results were obtained for treated cells and the other two replicates. Plots were generated using R [https://www.R-project.org/] and Rstudio [http://www.rstudio.com/]. ‘SSU peak coverage’ of mRNAs was defined as described for each gene: First, the highest peak of FPs in the 5′ UTR was identified and measured; second, the height of the AUG peak was measured; finally, the peak coverage was calculated by dividing the 5′ UTR FPs by the sum of the 5′ UTR and AUG FPs. Note that the data from three replicates was merged prior to plotting.

## RESULTS AND DISCUSSION

### A modified TCP-seq protocol for *S. pombe*

**Figure 1.**
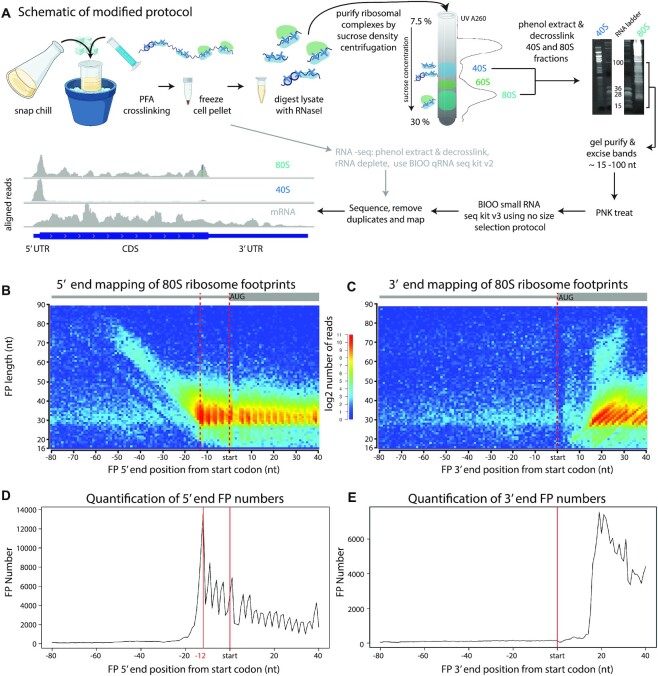
TCP-seq of 80S ribosomes. (**A**) Cartoon depicting the main steps of the optimised TCP-seq protocol (created with BioRender.com). *S. pombe* cells are snap chilled before cross-linking with formaldehyde. Cells are harvested and frozen. After lysis, cell extracts are treated with RNase I and separated by density centrifugation. Fractions containing either the 40S or 80S complexes are purified and decross-linked. FP RNAs are isolated on gels for library preparation and sequencing. (**B**) Metagene heatmap showing the relationship between FP length and FP location around the aligned initiation sites for 80S libraries. The distance between the 5′ end of the FP and the start codon is plotted. The colours indicate the number of FPs. (**C**) As in (A), but the distance to the 3′ end of the FP is plotted. (**D**) Projection of the data in B along the x-axis. (**E**) Projection of the data in (B) along the x-axis.

We optimised the TCP-seq protocol for *S. pombe* (Figure 1A, Materials and Methods). Cells were cross-linked with formaldehyde and subjected to mechanical lysis. The cell extracts were then treated with RNase I and sucrose gradient ultracentrifugation was used to separate 40S and 80S fractions (note that for simplicity we use 40S to refer to all small subunit complexes such as 43S and 48S). We introduced two key modifications to the original method. First, a simplification of the isolation of the sucrose gradient fractions. The original protocol involved two sequential sucrose gradients: the first one allowed the removal of free 60S ribosomal subunits, but also discarded mRNAs attached to single small subunits (SSUs) (7,32). We removed the first gradient from the protocol, thus obtaining a more unbiased view of the translatome. This change in the method also allowed a reduction of the amount of starting material, making the protocol more efficient. Second, we introduced the use of Unique Molecular Identifiers (UMIs) for library preparation. UMIs allow better quantification of sequencing libraries by removing PCR amplification biases and, therefore, lead to the production of a non-redundant sequence dataset (45,46).

We applied the modified TCP-seq protocol to *S. pombe* cells growing in minimal medium (EMM2) before and after treatment with the histidine analogue 3-amino-triazol (3-AT), which blocks histidine biosynthesis and thus causes amino acid starvation. To ensure consistency in the results, we performed 3 independent biological replicates for each of the two conditions (Supplementary Figure S1A). In addition, to increase the coverage of the translatome, we generated several independent 40S and 80S libraries from each biological replicate, which were processed in parallel and pooled for analysis (see Materials and Methods).

We initially explored the overall behaviour of SSUs and full translating ribosomes to validate our protocol. As rRNA depletion was not performed, the amount of different rRNA species can be used to estimate the enrichment and purity of the purified fractions. 80S-derived libraries contained 28S and 18S rRNAs in roughly equimolar proportions, whereas 40S-based libraries were very strongly enriched in 18S rRNAs and contained very small amounts of 28S (Supplementary Figure S1B). Moreover, FPs from 80S fractions mapped preferentially to initiation sites and coding sequences, whereas FPs from 40S libraries were heavily enriched at initiation sites and 5′ UTRs (Supplementary Figure S1C). Both sets of libraries contained very small amounts of FPs mapping to termination codons and 3′ UTRs (Supplementary Figure S1C). A fraction of 40S FPs mapped to coding sequences. A similar observation has been reported in previous TCP-seq experiments (32) and is thought to be caused by 60S subunits poorly cross-linked to the rest of the ribosome that become detached during the purification process. Consistent with this interpretation, 40S FPs that map to coding sequences tend to have lengths similar to 80S-protected footprints (Supplementary Figure S2) and display triplet periodicity (Figure 1B, FPs located in the CDS). Overall, these results demonstrate that our optimised TCP-seq protocol specifically captures 40S SSUs and 80S ribosomes.

**Figure 2.**
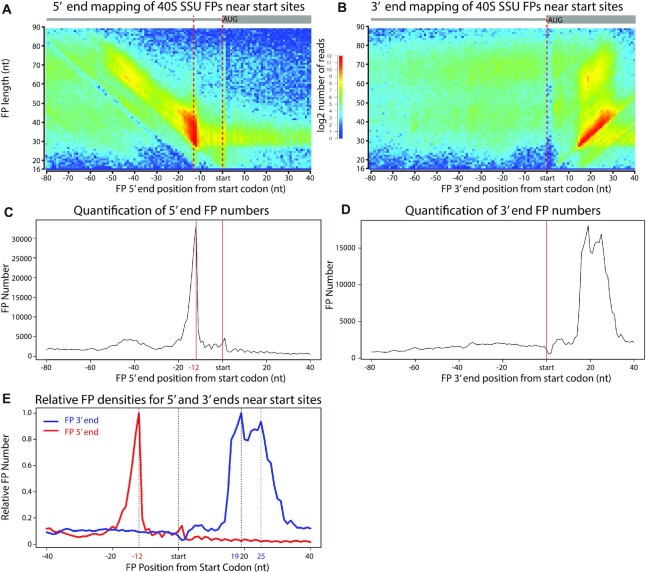
TCP-seq of 40s complexes. (**A**) Metagene heatmap showing the relationship between FP length and FP location around the aligned initiation sites for 40S libraries. The distance between the 5′ end of the FP and the start codon is plotted. The colours indicate the number of FPs. (**B**) As in (A), but the distance to the 3′ end of the FP is plotted. (**C**) Projection of the data in (A) along the x-axis. (**D**) Projection of the data in (B) along the x-axis. (**E**) Data for (C) and (D) plotted on the same graph to facilitate their comparison.

FPs from both 40S and 80S libraries displayed a wide range of lengths. We examined the size distribution of FPs that mapped to different features of mRNAs (Supplementary Figure S2). Outside 5′ and 3′ UTRs, 80S libraries showed well-defined peaks around 32 nucleotides. These FPs resembled those of standard ribosome profiling, albeit slightly longer (32). FPs from 5′ and 3′ UTRs also had shorter lengths, but they were not abundant (Supplementary Figure S2). By contrast, 40S libraries displayed extended protection over two separate peaks, particularly on initiation sites and 5′ UTRs (Supplementary Figure S2, see below). Generally, treatment with 3-AT did not alter the characteristics of 40S or 80S complex protected FPs on a genome-wide scale (Supplementary Figure S2).

### Translation complexes at the initiation site and the 5′ UTR

Both 80S and 40S FPs were enriched at initiation codons (Supplementary Figure S1C). 80S FPs showed a single peak of length of 31–35 nucleotides (Supplementary Figure S2), with the exact size varying among the biological replicates (not shown). 40S FPs displayed a bimodal pattern of protection, with a major peak at around 32 nucleotides and a distinct second peak around 65 nucleotides (Supplementary Figure S2). Moreover, the 32-nucleotide peak was broader in the 40S than in the 80S libraries (Supplementary Figure S2). Thus, the length of both populations of mRNA protected by SSUs is clearly longer than that that of fragments protected by a full ribosome (Supplementary Figure S2).

Different populations of footprints likely represent different initiation complexes. These complexes are defined by their position on the mRNA as well as by the length of the mRNA fragment they protect (i.e. the FP length). To investigate the properties of initiation complexes, we used heatmaps to visualise the density of FPs of given lengths (y axis) versus their distance of the FP to the initiation codon (x axis). As different FP ends can behave differently, we mapped the 5′ and 3′ ends of the FPs separately (Figures 1B, C and 2A, B). Projections of the data along the x axes of the heatmaps, representing distance from the initiation codon to the 5′ end or the 3′ end of the FP for fragments of all lengths, are shown (Figures 1D, E and 2C, D, E). Supplementary Figure S3 helps with the interpretation of the plots.

When the 5′ ends of the 80S FPs were plotted relative to the distance to the initiation codon, 80S ribosomes accumulated around position –12 (Figure 1B, D). This enrichment corresponds to a ribosome with the P site in position 0 (the A of the AUG codon) and is consistent with standard ribosome profiling data. As expected, FPs from 80S libraries showed periodicity in the coding sequences (enrichment of every third nucleotide along the coding sequence) and were relatively rare in 5′ UTRs (Figure 1B, D). The weak diagonal line of longer 80S FPs is likely to represent 5′ extensions of the FPs, as 3′ end mapping of these reads aligns to the same position (Figure 1B, C). This additional protection is likely conferred by lingering eIFs.

FPs from 40S libraries (mapped at the 5′ end) also accumulated at position –12 but displayed a much stronger diagonal line of longer FPs, suggestive of widespread protection by eIFs (or possibly queuing of SSUs upstream of the initiation codons, see Figure 2A, C). Most complexes at the initiation site showed a common 5′ end (vertical line at –12 in the 5′ mapped FPs, Figure 2A, C) of variable lengths (diagonal line in the 3′ mapped FPs, Figure 2B, D). We investigated this further by plotting 5′ and 3′ end FP densities against their distance from the start codon for a metagene (Figure 2E). Consistent with the heatmap, the distribution of SSU initiation complexes showed a single sharp 5′ peak at –12 (red line), and two overlapping peaks at the 3′ (at +19 and +25, blue line). Thus, our data reveal the presence of at least two separate SSU complexes at the start codon.

In budding yeast, TCP-seq identified 3 major SSU complexes, with a common 5′ position (–12 from the initiation codon) and 3′ ends centred around positions +6, +16 and +24 (35). Mammalian SSUs also showed a single 5′ end location (-12 to -14) and less well-defined positions at the 3′ (a very small peak at +5/+8 and overlapping peaks at +15/+18 and +23/+25) (10)]. Therefore, the *S. pombe* distribution of FPs, with both major peaks overlapping, appears to be more similar to that of humans, possibly reflecting similarities between these two organisms that are not shared with *S. cerevisiae* (such as the more similar composition of eIF3) (27) attributing to different conversion states from 40S to 80S (10).

### Cap recognition and scanning of the 5′ UTRs

We also interrogated the 5′ and 3′ ends of FPs aligned to the annotated transcription start site of the mRNA, as experimentally determined by Cap Analysis of Gene Expression (CAGE) (Supplementary Figure S4) (42). As expected, SSU FPs were very rare upstream of the mapped transcription start site. The 5′ end of FPs accumulated evenly from the 5′ end of transcripts, starting from the transcription start site (Supplementary Figure S4A, C, reads accumulate at the transcription start site as a vertical line). These FPs covered the whole range of detectable sizes. The 3′ end of these FPs showed variable positions (Supplementary Figure S4B, D, diagonal line of accumulated reads from the transcription start site), indicating that the FPs from SSUs binding to the transcription start site have variable 3′ ends. This type of alignment is similar to observations in vertebrates (fish) and yeast (33); however, FPs in *D. rerio*, and to a lesser extent in *S. cerevisiae*, accumulated more sharply exactly at the 5′ mRNA start. As *S. pombe* lack eIF3k/l proteins predicted to interact with the 5′-CAP (47), this difference may reflect alternative protein-RNA interactions and kinetics

### Gene-specific translational control

**Figure 3.**
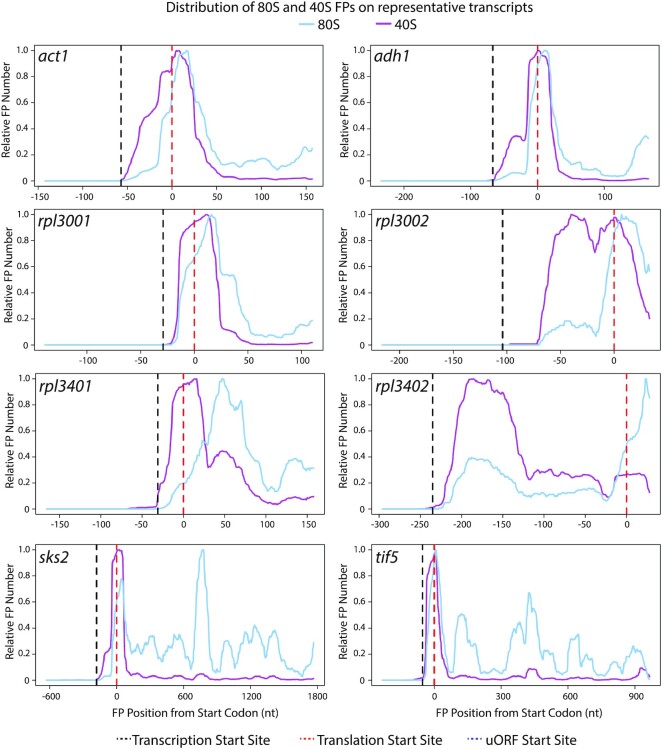
Distribution of 80S and 40S FPs for eight selected transcripts. Normalised FP densities for individual mRNAs at the 5′ UTRs and translation initiation sites. X-axes: distance to the initiation codon; transcription start sites (black dashed lines); translation initiation sites for main coding sequence (dashed red lines); AUG-starting uORFs (dotted blue lines). Y-axes: relative FP number, obtained by normalising 80S/40S FP numbers by their corresponding maximum value (80S blue lines, 40S purple lines). Note the whole length of the FPs is plotted (cf. Figures 1 and 2 where only 5′/3′ ends are plotted).

**Figure 4.**
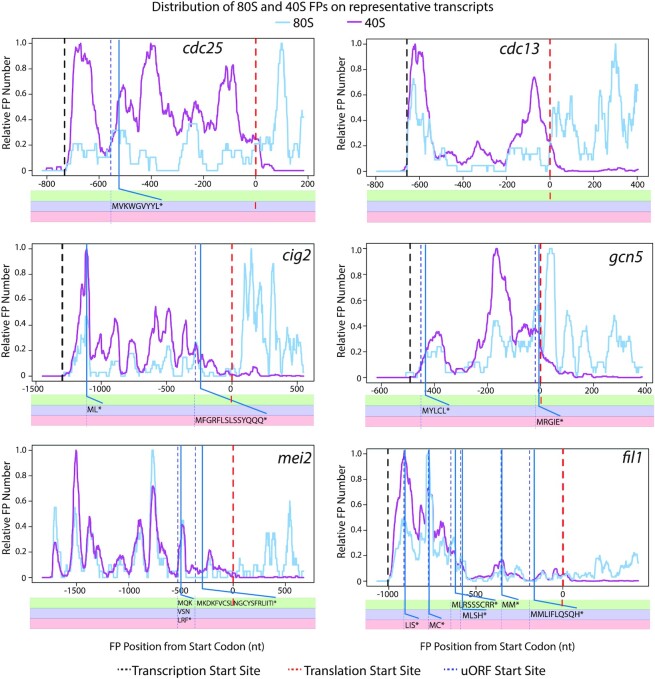
Distribution of 80S and 40S FPs for six selected transcripts. Normalised FP densities for individual mRNAs at the 5′ UTRs and translation initiation sites. X-axes: distance to the initiation codon; transcription start sites (black dashed lines); translation initiation sites for main coding sequence (dashed red lines); AUG-starting uORFs (dotted blue lines). Y-axes: relative FP number, obtained by normalising 80S/40S FP numbers by their corresponding maximum value (80S blue lines, 40S purple lines). Note the whole length of the FPs is plotted (cf. Figures 1 and 2 where only 5′/3′ ends are plotted). All data are for replicate two except for *gcn5*, which shows aggregated data for three replicates.

Visual inspection of the distribution of 40S and 80S FPs in individual genes revealed a rich diversity of patterns, from single peaks at initiation sites to complex patterns of accumulation of FPs on 5′ UTRs (Figures 3 and 4). To explore different modes of regulation, we defined the ‘SSU peak coverage’ of an mRNA as a measure of the accumulation of SSUs in 5′ UTRs compared to initiation sites (see Supplementary Figure S5 for definition and plots). mRNAs were then ranked based on their SSU peak coverage (Supplementary Figure S5). Figures 3 and 4 show the distribution of 40S/80S FPs for 14 representative genes with different patterns of SSU peak distribution.

Transcripts with low SSU peak coverage (Supplementary Figure S5, below 0.2) tended to accumulate both 40S and 80S FPs at initiation sites, often with a shoulder towards the 5′ direction (Figure 3, *act1, adh1*, *rpl3001* and*, rpl3401*). This suggests that events at initiation sites (and not scanning of the 5′ UTR) are limiting for initiation. This group of transcripts included highly-expressed genes, some of them encoding cytoskeletal proteins (*act1*, encoding actin) or metabolic enzymes (*adh1*, encoding alcohol dehydrogenase). Low SSU peak coverage genes also encompassed many genes encoding ribosomal proteins (such as *rpl3001* and *rpl3401*), which usually have very short 5′ UTRs and a single SSU peak at the initiation site. Very few ribosomal protein genes had high peak coverage, with some notable exceptions: the ribosomal protein genes *rpl3002* and *rpl3402* displayed a SSU peak in their 5′ UTRs, close to the initiation sites (Figure 3, *rpl3002* and *rpl3402*). Interestingly, both genes had paralogues (*rpl3001* and *rpl3401*, respectively, see above) with very low peak coverage. This suggests that both paralogues are independently regulated at the translational level, possibly indicating specialised functions.

**Figure 5.**
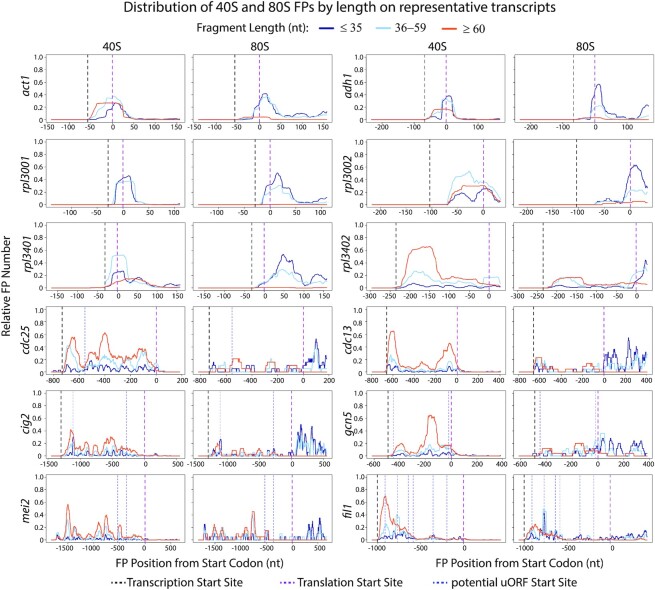
Distribution of 80S and 40S FPs of different lengths for 12 selected transcripts. Data from Figure 3 and Figure 4 segmented by FP lengths. X-axes: distance to the initiation codon; transcription start sites (black dashed lines); translation initiation sites for main coding sequence (dashed red lines); AUG-starting uORFs (dotted blue lines). Y-axes: Relative FP numbers were obtained by normalising 80S/40S FP numbers by their corresponding maximum value (all lengths): FP length ≤35 nucleotides (dark blue), FP length 36–59 (light blue), FP length ≥60 (orange). Note the whole length of the FPs is plotted (cf. Figures 1 and 2 where only 5′/3′ ends are shown). All data are for replicate 2 except for *gcn5*, which shows aggregated data for three replicates.

At the other end of the scale, an interesting set of genes encoding key regulators of cellular function have long 5′ UTRs with large regions of high SSU coverage (and occasional 80S peaks), sometimes without forming discrete peaks and in parts of the 5′ UTR devoid of uORFs (Figure 4). This group includes *cdc25* (a key cell cycle regulator), *cdc13* and *cig2* (encoding cyclins) and *mei2* (an RNA-binding protein that promotes meiosis entry). A gene that has been reported as translationally regulated by 3-AT (*gcn5*) (48) also showed clear SSU peaks outside initiation sites. Finally, we identified *fil1* as one of the genes with highest SSU peak coverage. Surprisingly, the majority of SSU FPs in 5′ UTRs were not associated with AUG-initiation sites (Figures 4 and 5, *fil1*). A detailed discussion of *fil1* regulation in the context of amino acid starvation is presented below.

FPs of different lengths represent complexes involved in different stages of translation initiation. We investigated the distribution of FPs of different lengths on individual genes by binning FPs into three groups: shorter than 36 nucleotides (which roughly correspond to the protection given by a full elongating ribosome), between 36 and 59 nucleotides (inclusive), and larger than 59 nucleotides (Figure 5, dark blue, light blue and orange data respectively). Overall, 80S libraries contained mostly shorter FPs that mapped to initiation sites and coding sequences, whereas 40S libraries were enriched in longer fragments located at initiation sites and 5′ UTRs.

40S libraries of highly expressed genes, such as *act1* and *adh1*, were mostly enriched in short FPs around the initiation sites. A fraction of their FPs displayed extended protection towards the 5′ end of the initiation sites (see orange peak extending towards the 5′ side). Many ribosomal protein genes (*rpl3001, rps1001*) that showed a single peak of footprints at the initiation site did not display any protection of longer fragments, possibly due to the short length of their 5′ UTRs. By contrast, longer footprints were predominant across longer 5′ UTRs (see *cdc25*, *cdc13*, *cig2* and *mei2*) (Figure 5).

SSUs in initiation sites generally protect smaller RNA fragments, with longer protection probably representing lingering initiation factors or queueing SSUs. SSUs in 5′ UTRs have more extended FPs, and likely represent scanning subunits. Overall, our data identify numerous FP accumulation sites (Figure 4) that may modulate SSU movement along transcripts, by mechanisms such as non-AUG-mediated initiation of uORFs (49) or RNA secondary structures (50).

### Transcriptomic responses to amino acid starvation

We have previously investigated the transcriptomic response of fission yeast to amino acid starvation using RNA-seq. To validate the TCP-seq experimental setup, we performed RNA-seq of the 6 biological samples used in this work (plus/minus 3-AT and three biological replicates of each of them). The samples were aliquots of the cross-linked material used for TCP-seq (Figure 1A). We identified differentially expressed genes (induced or repressed upon stress) using Deseq2 (43). A total of 200 genes were significantly up-regulated and 182 down-regulated (adjusted *P*-value < 0.01 and minimal change of 2-fold) (Supplementary Table S1). Up-regulated genes were enriched in genes involved in amino acid metabolism, whereas down-regulated genes were enriched in translation-related processes, including genes encoding translation factors and ribosomal proteins. Many of these genes are transcriptionally down-regulated in response to multiple stress conditions, as part of the so-called core environmental stress response (CESR) (51). Both the up- and the down-regulated categories identified in this study were very similar to those found in our previous standard RNA-seq analyses of this response (Supplementary Figure S6 (21)). Overall, the RNA-seq results validate our TCP-seq approach and demonstrate that the process of formaldehyde fixation and collection of cells for TCP-seq does not cause major changes in the transcriptome.

### Translational control in response to amino acid starvation

To investigate global effects on translation caused by 3-AT treatment, we plotted SSU and ribosome average densities (metagenes) around translation initiation sites (Supplementary Figure S7 and data not shown for other replicates). This analysis did not reveal any clear differences in the shapes of the metagenes, consistent with the idea that 3-AT reduces the translation of the majority of transcripts to the same extent. We then focused on individual genes and mined the TCP-seq dataset to identify genes translationally regulated by amino acid starvation. The translational efficiency of a transcript (TE) was defined as the number of 80S ribosomal FPs normalised by the transcript mRNA levels (we only used RNA-seq reads that mapped to coding sequences). We used a bespoke tool (ΔTE (44)) to identify genes with changes in TE upon 3-AT treatments (see Materials & Methods). Only one gene (*fil1*) showed significant upregulation and 42 genes were significantly down-regulated (adjusted *P*-value < 0.01 and minimal change of 2-fold) (Supplementary Figure S8A). The latter group was enriched in genes involved in translation, including translation factors, ribosomal proteins and tRNA ligases (Supplementary Table S1). Many of the 42 genes had small 5′ UTRs and a single peak of 40S accumulation at the AUG of the main coding sequence. They did not show any clear change in the distribution upon 3-AT treatment that suggests a possible mechanism of the repression (*sks2* and *tef5*, Figure 3).

### Translational control of the *fil1* gene

**Figure 6.**
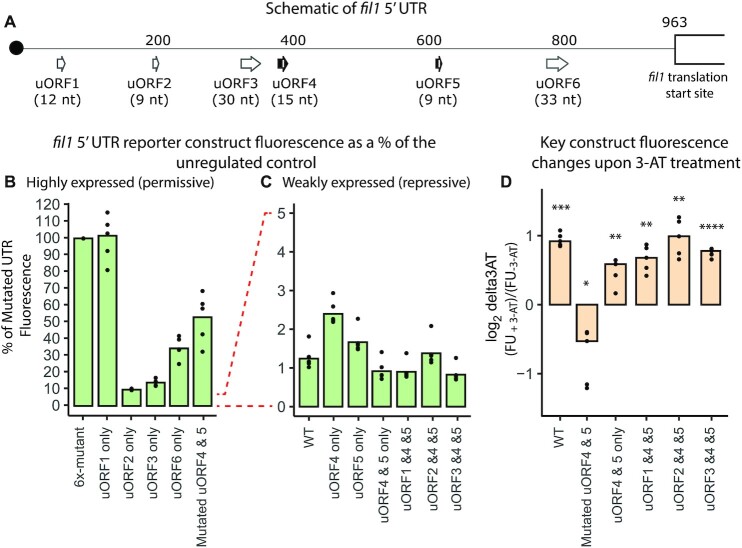
Reporter analysis of the *fil1* 5′ UTR. (**A**) Schematic of the *fil1* 5′ UTR showing the location and length of six uORFs. (**B**) Fluorescence levels of reporters containing highly-expressed versions of the *fil1* 5′ UTR in the absence of 3-AT. Data have been normalised to the fluorescence levels of the construct with all uORFs mutated (100%, 6x-mutant). (**C**) As in B, but showing the data from weakly-expressed constructs in the absence of 3-AT. Data normalised as in (B). (**D**) Changes in fluorescence reporter levels expressed as the ratio between 3-AT-treated and untreated control cells (log_2_-transformed). Data are from five independent biological replicates (dots represent the values from each experiment and bars show the corresponding means. Statistical significance was determined using paired *t*-tests: * *P* ≤ 0.05; ** *P* ≤ 0.01; *** *P* ≤ 0.001; **** *P* ≤ 0.0001.

We have previously shown that *fil1* translation is regulated by its 5′ UTR, which contains six translated uORFs (uORF1, with a non-AUG start codon, and uORF2-6, each starting with AUG) (Figure 6A shows uORF distribution and lengths). Translation of the endogenous *fil1* gene is up-regulated 3.8-fold upon 3-AT treatment, and this is accompanied by increased levels of the endogenous Fil1 protein (21).

To investigate the role of uORFs in *fil1* regulation, we generated a fluorescent reporter system under the control of a constitutive promoter (*adh1* gene) and different variations of the 5′ UTR of the *fil1* gene. Reporter genes did not show changes in RNA levels in any of the experiments, so changes in fluorescence of the reporters discussed below most likely reflect reporter mRNA translation (Supplementary Figure S9A, D). We initially examined the effects of the mutations on reporter translation in the absence of stress (Figure 6B, C and Supplementary Figure S9A, B). A reporter gene under the control of a mutated version of the *fil1* 5′ UTR in which all six uORFs have been inactivated by mutating their initiation codons (6x-mutant) displayed high levels of translation. By contrast, the fully regulated reporter with the wild type (WT) *fil1* 5′ UTR has very weak translation, only 1.16% of the unregulated 6x-mutant (Figure 6B, C, Supplementary Figure S9A, B). To investigate the role of individual uORFs, we generated mutant versions of the reporter system in which only one uORF was active and normalised the data to the levels of the 6x-mutant reporter. Using this system, uORF4 and uORF5 were highly inhibitory, with reporter level translation close to that of the WT reporter (at 2.2% and 1.6% of the 6x-mutant). The other 4 reporters displayed translation levels of 9–97% of those of the 6x-mutant (Figure 6B, C, Supplementary Figure S9A, B).

We then looked at the ability of the reporters to respond to 3-AT. Whereas the WT reporter nearly doubled in translation, the 6x-mutant reporter translation was down regulated in response to 3-AT treatment (Figure 6D, Supplementary Figure S9C). This is consistent with the model that translational control of the *fil1* gene is mediated by the uORFs in its 5′ UTR (21) and that 3-AT induces general translational repression. The results suggest that the translational control of *fil1* could be based on the combination of ‘repressive’ uORFs (uORF4 and uORF5), which do not favour reinitiation, and ‘permissive’ uORFs that promote reinitiation and/or support leaky scanning. We first investigated the ability of single uORFs to respond to 3-AT (Supplementary Figure S9C). uORFs 1, 2, 3 and 6 were down-regulated by 3-AT, probably reflecting the overall decrease in translation caused by 3-AT. However, uORFs 4 and 5 (the repressive ones) did not show strong changes in absolute levels. This suggests that a single repressive uORF can sustain some response to 3-AT, but not reaching the levels of wild type. Moreover, a construct containing only uORFs 4 and 5 showed responsiveness to 3-AT, but not as strong as the wild type (Figure 6D and Supplementary Figure S9C). We then looked at whether the addition of an upstream permissive uORF to the uORF4/5 would allow it to behave as wild type (Figure 6A, D and Supplementary Figure S9C). Indeed, combinations of active uORFs 1/4/5, 2/4/5 and 3/4/5 behaved very similar to the wild type reporter. Additionally, mutating any of the six uORFs individually only modestly effected translation and response to 3-AT (Supplementary Figure S9A–C) indicating that as with *S. cerevisiae GCN4*, there is redundancy built into *fil1* translational regulation. We conclude that a combination of permissive and repressive uORFs are necessary for full regulation of *fil1* translation. In the wild type reporter, one or more permissive uORFs would capture the scanning SSUs and allow reinitiation. In the absence of a permissive upstream uORF, a non-AUG initiation event might occur with low efficiency. This model is consistent with the TCP-seq data discussed below.

**Figure 7.**
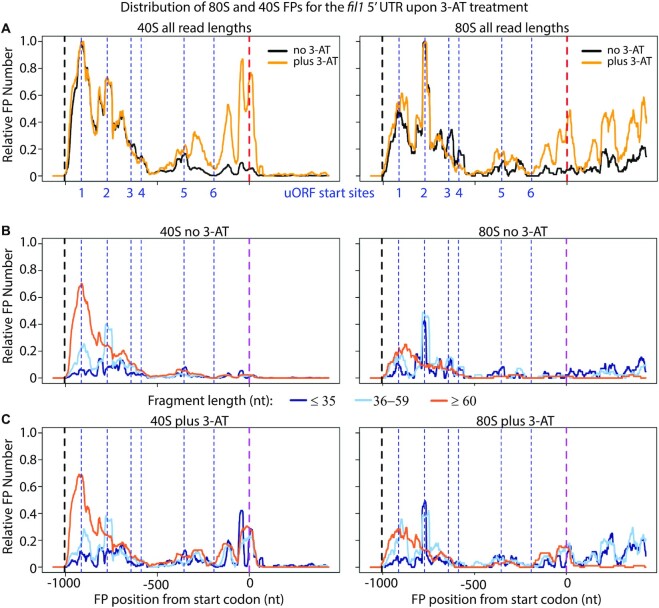
Distribution of 80S and 40S FPs on the *fil1* gene upon 3-AT treatment. (**A**) Normalised FP densities for the *fil1* mRNA at the 5′ UTR and translation initiation sites. X-axis: distance to the initiation codon; transcription start sites (black dashed lines); translation initiation sites for main coding sequence (dashed red lines); uORFs (dotted blue lines); uORF1 starts with CUG and uORF 2–6 with AUG. y-axis: relative FP number, obtained by normalising 80S/40S FP numbers by their corresponding maximum value. Cells were untreated with 3-AT (black) or treated (yellow). (**B**) As in (A), but with data segmented by length; translation initiation sites for main coding sequence (dashed purple line). FP length ≤35 nucleotides (dark blue), FP length 36–59 (light blue), FP length ≥60 (orange). Note the whole length of the FPs is plotted (cf. Figures 1 and 2 where only 5′/3′ ends are shown). All data are for replicate 2. (**C**) As in (B), but for 3-AT-treated cells.

We then examined the distribution of SSUs in the TCP-seq datasets (Figure 7). In normally growing cells, uORF1 accumulated high levels of SSUs, which decreased slightly as they traversed uORF2 and uORF3. These uORFs also contained 80S ribosomes, consistent with the idea that they are translated. By contrast, uORF4, uORF5 and uORF6 had very few SSUs and low levels of 80S ribosomes (Figure 7A). These distributions are consistent with the repressive activities of uORF4 and uORF5 seen in the reporter experiments, with these two uORFs capturing the majority of the SSUs. Strikingly, SSUs accumulated downstream of uORF4 in cells treated with 3-AT (Figure 7A), showing that in these cells the repressive activities of uORF4 and uORF5 are overridden by the scanning SSUs. We quantified these changes in the distribution by measuring the fraction of 40S FPs on the second half of the 5′ UTR (nucleotides 720–1200, containing uORFs 4–6). Treatment with 3-AT caused an increase in FPs in the second half of 3.8-fold (paired *t*-test, *P* = 0.007) (Supplementary Figure S8B).

We examined the behaviour of SSUs of different lengths in the *fil1* 5′ UTR by partitioning SSUs into three groups as described above: up to 35 nucleotides, 36–59 nucleotides (inclusive), and longer than 59 nucleotides (Figure 7B, C, dark blue, light blue and orange lines respectively). The longest group of FPs was prevalent at the beginning of the mRNA and peaked at uORF1, presumably representing full scanning subunits. The middle-FP group showed peaks at uORF1-2, corresponding to earlier stages of initiation complex. Finally, short FPs showed multiple peaks, but were present at low levels (Figure 7B). Treatment with 3-AT did not affect much the distribution of FPs upstream of uORF4 but caused the appearance of peaks containing FPs of all 3 group lengths, with the shortest ones now being prevalent (Figure 7C).

In a simple model, analogous to the *GCN4*/*ATF4* paradigm, uORF1-3 would be translated but allow reinitiation to take place to relatively high levels. Translation of uORF4 and 5 causes full termination and prevents reinitiation. In the presence of 3-AT, decreased levels of TC would allow scanning SSUs to bypass repressive uORFs 4 and 5, and translation of *fil1* may occur if a new TC is acquired. The role of uORF6 in the system is unclear, and it might be constitutively by-passed by leaky scanning.

Although TCP-seq can provide unprecedented views of the *in vivo* translational landscape of a cell, it has only been applied to a handful of systems and conditions (10,11,32,35). The results we present here demonstrate that TCP-seq captures the dynamic changes in translation initiation under stress and shows how this information provides *in vivo* mechanistic insight into regulatory mechanisms.

## DATA AVAILABILITY

All sequencing files have been deposited in ArrayExpress, with accession number E-MTAB-12264 (52).

## Supplementary Material

gkac1140_Supplemental_Files
