# Supplementary material for: Translation-complex profiling of fission yeast cells reveals dynamic rearrangements of scanning ribosomal subunits upon nutritional stress
Source: Nucleic Acids Res. 2022 Dec 8;50(22):13011–25. doi: 10.1093/nar/gkac1140 (PMC9825154; doi:10.1093/nar/gkac1140)
Supplement: gkac1140_Supplemental_Files [file gkac1140_supplemental_files.zip › supplemental_figures_Duncan_Mata.pdf]

Supplemental Figures

Translation-complex profiling of fission yeast cells reveals dynamic rearrangements of scanning ribosomal subunits upon nutritional stress

Caia Deborah Suzanne Duncan and Juan Mata *

Department of Biochemistry

University of Cambridge

Cambridge CB2 1QW

United Kingdom

* To whom correspondence should be addressed jm593@cam.ac.uk

This PDF contains Figures S1-S9

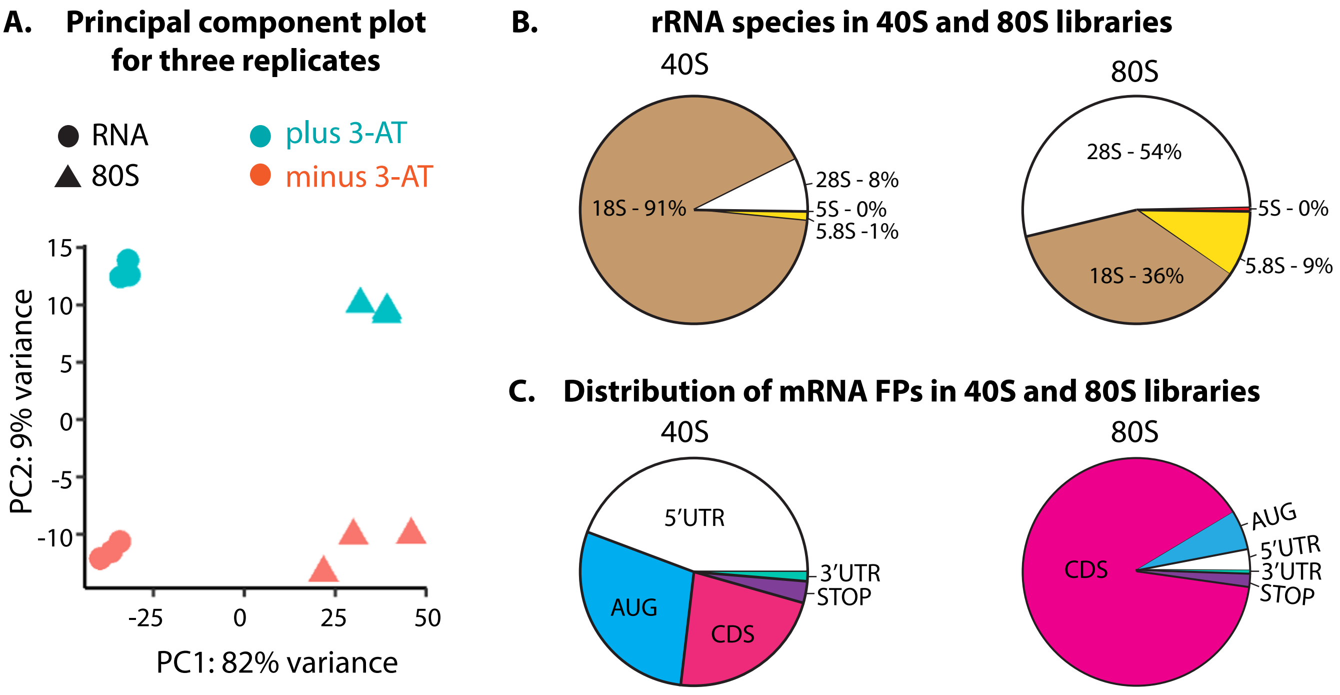

Duncan & Mata (2022), Supplemental Figure 1

Figure S1. Overview of the TCP-seq experiments. A. Principal Component Analysis (PCA) of 3 independent replicates of TCP-seq (80S) and their correspondent RNA-seq experiments (RNA). Data are shown for untreated cells (minus 3-AT) and treated cultures (plus 3-AT). As expected, replicates for similar samples cluster together. **B.** Distribution of rRNA fragments in 40S and 80S libraries. Pure 40S SSU samples contain 18S rRNA, whereas 80s ribosomes contain have 5S, 5.8S, 18S and 28S. **C.** Distribution of FPs to different mRNA features for 40S and 80S libraries.

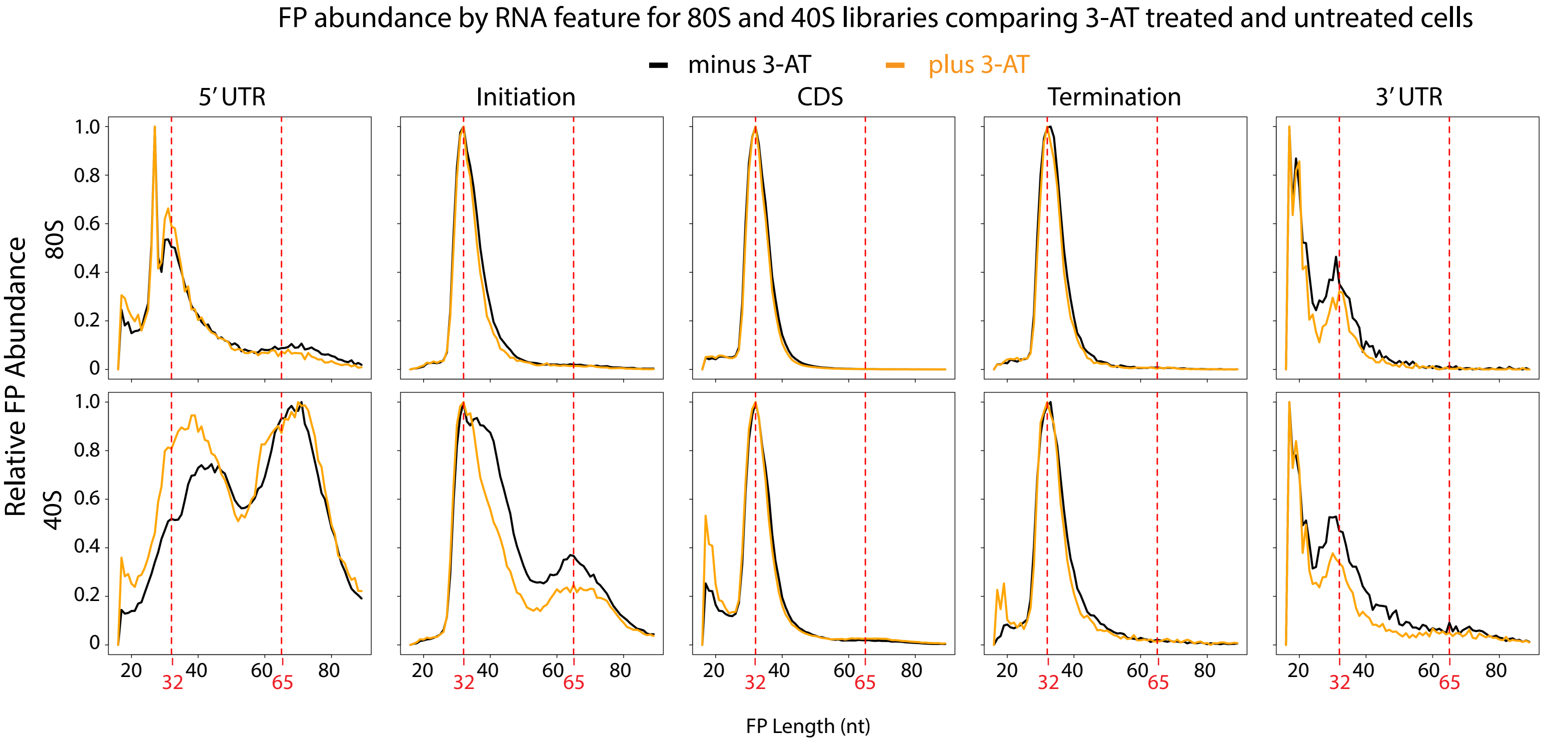

Duncan & Mata (2022), Supplemental Figure 2

Figure S2. Distribution of footprint (FP) lengths for 40S and 80S libraries across different transcript features. Data are shown for control (black) and 3-AT treated cells (orange). Red dotted lines at 32 and 65 nt highlight the centre of peak densities across all libraries. Relative FP number, obtained by normalising 80S/40S FP numbers by their corresponding maximum value.

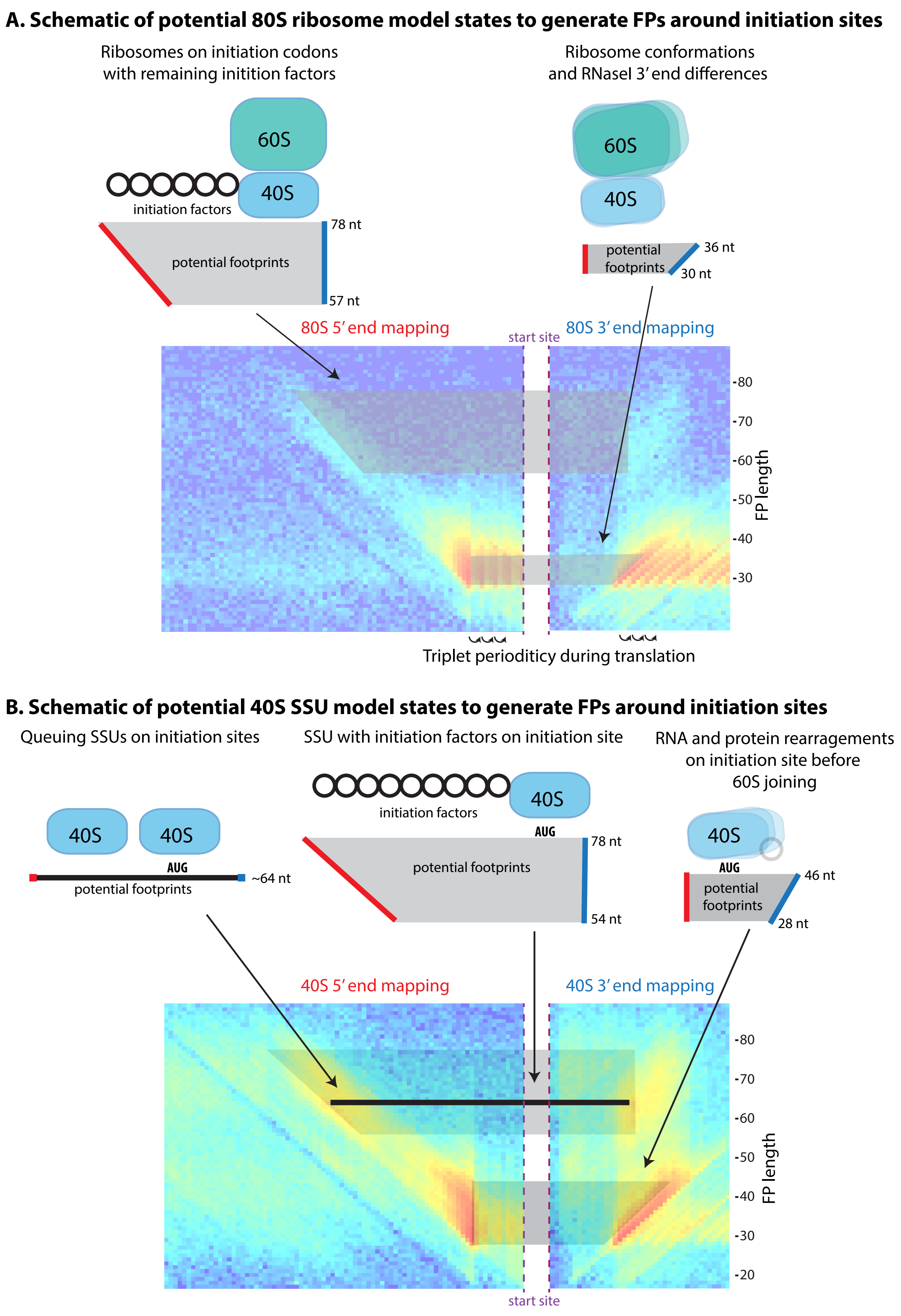

Duncan & Mata (2022), Supplemental Figure 3

Figure S3. Schematics of potential ribosome and SSU model states to generate FPs around translation initiation sites. A. Related to Figure 1, 80S-generated FPs. FPs produced from fully assembled ribosomes centre around 30-36 nt with sharp 5’ ends (right cartoon, red line) and variable 3’ ends (right cartoon, black line). This pattern is also seen in standard ribosome profiling, and is likely due to accessibility differences of the RNase I to the 3’-end of the FP. Elongating ribosomes decode mRNA codon by codon, giving both the 5’ and 3’ end mapping data a distinctive 3 nt periodicity in the CDS (heatmap). During ribosome assembly many initiation factors dissociate or rearrange their association with the 40S before later release. As many eIFs are predicted to associate on the 5’ end of the SSU(49), we anticipate longer FPs (~57-78nts) with heterogenous 5’ ends mapping further from the start site (red bar in the left cartoon) and sharp 3’ ends (blue bars in the left cartoon). The arrows indicate the location of different FPs on the heatmaps. **B**. Related to Figure 2, 40S-generated FPs. Longer FPs generated from SSU protection are likely due to eIFs contacting mRNA. Before 60S recruitment, most eIFs dissociate from the complex, many undergoing rearrangements during this process. The majority of SSU FPs mapping to start sites are between 28-46 nt long, with a sharp 5’ end (red line in the right cartoon) and heterogenous 3’ ends (black line in the right cartoon), which indicate different complexes are being captured. Longer FPs (54-78 nt, middle cartoon) may indicate 40S in complex with initiation factors (similar to those for 80S, see above). It is also possible that longer fragments (centred around 64 nt) correspond to queuing SSUs (left cartoon). However, it is unknown if scanning in *S. pombe* is 5’ CAP-tethered or CAP-severed; if severed, there is the potential for SSUs to queue at rate limiting stages of scanning. Faint triplet periodicity (as seen in the 80S data above) indicates dissociation of weakly cross-linked 60S from translating ribosomes in 40S libraries.

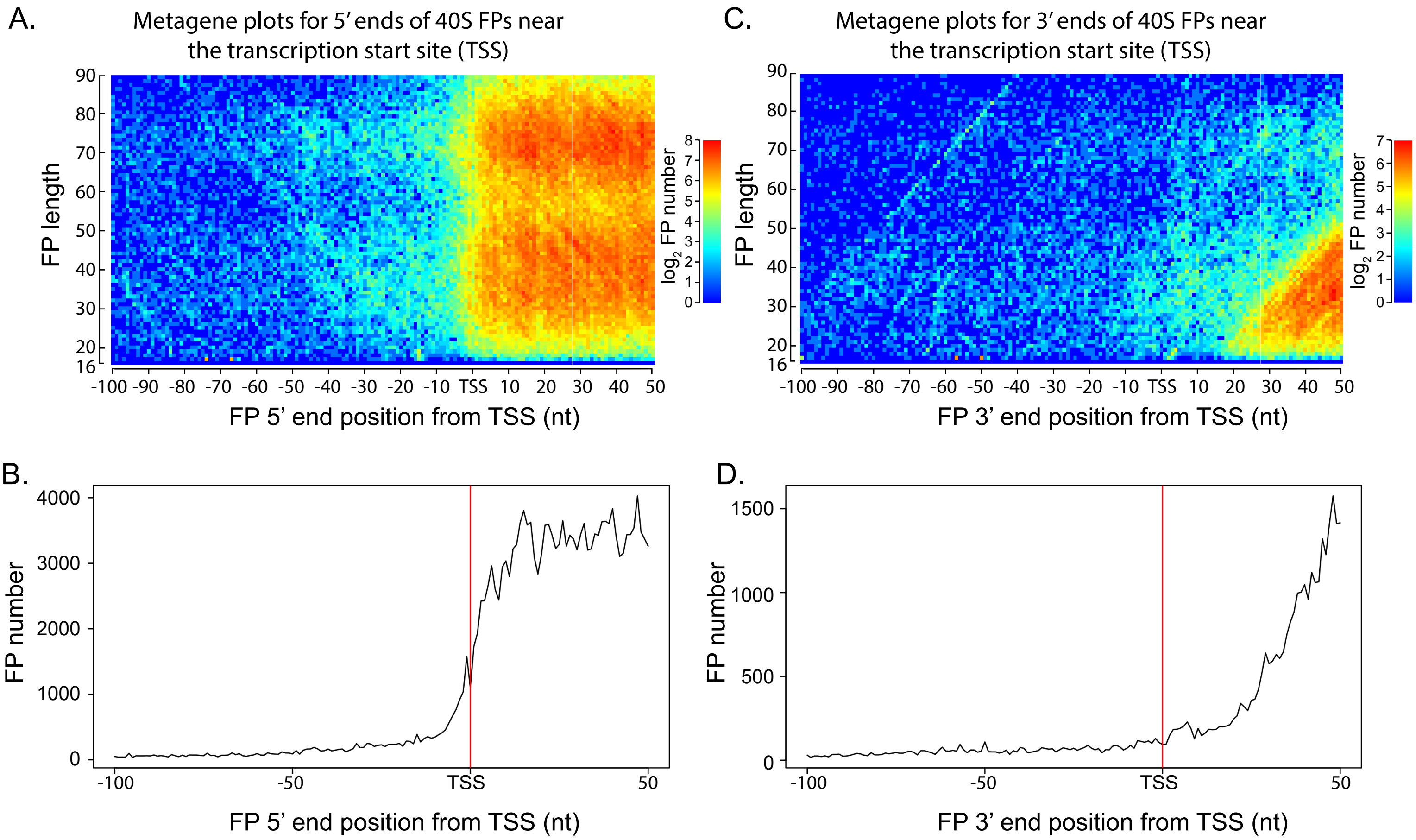

Duncan & Mata (2022), Supplemental Figure 4

Figure S4. TCP-seq of transcription start sites. A. Heatmap showing a metagene plot of footprint (FP) length and FP location around the transcription start site for 40S libraries not treated with 3-AT. The distance between the 5' end of the FP and the start codon is plotted. Only genes with 5' UTRs longer than 100 nt were analysed. The colours indicate the number of FPs. **B.** Projection of the heatmap in A along the x-axis. **C.** As in A, but the distance to the 3' end of the FP is plotted. **D.** As in B for 3'-end positions.

Ranking of genes by increasing ratio of 5'UTR peak to start codon peak for untreated and 3-AT treated cells

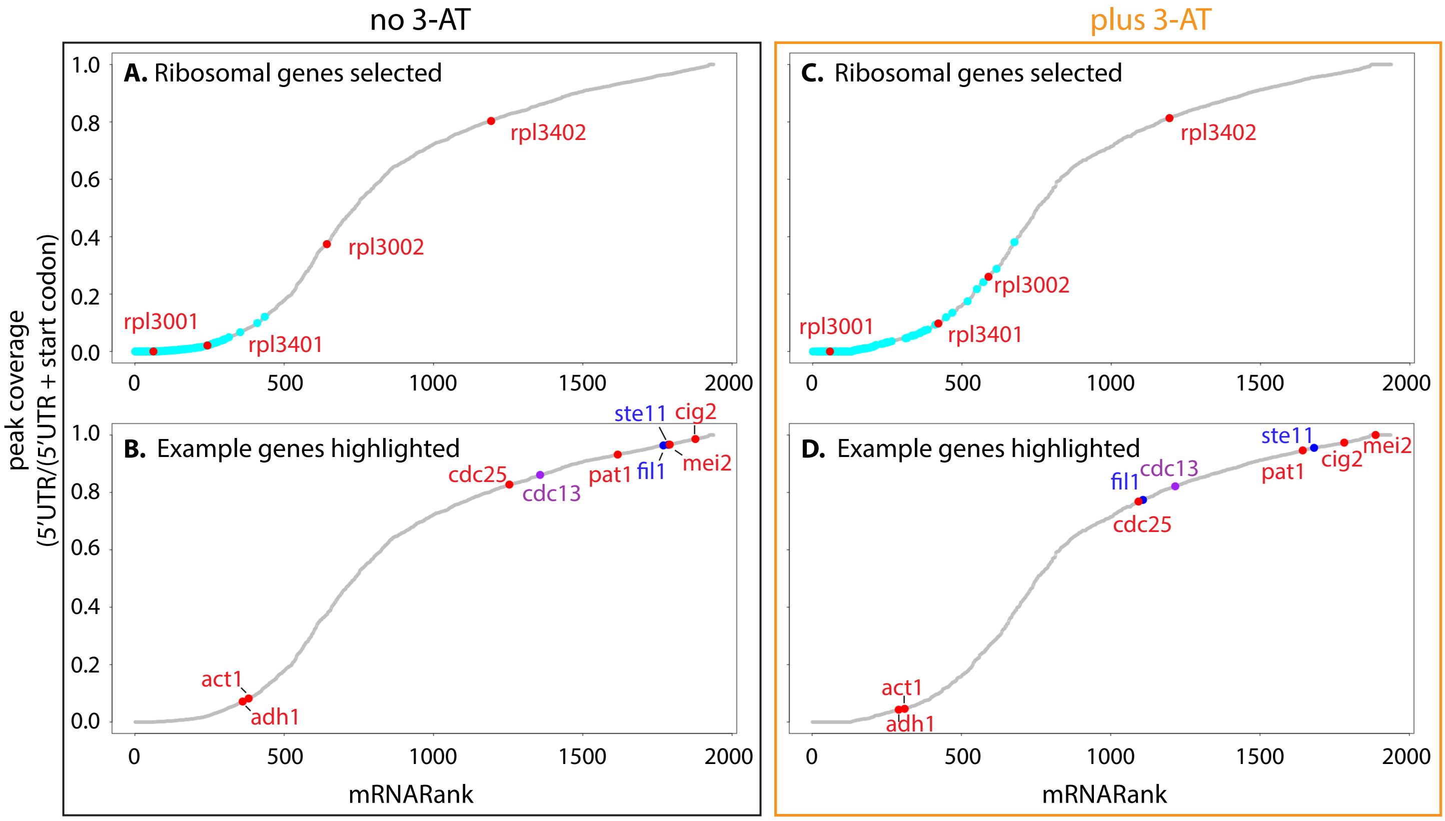

Duncan & Mata (2022), Supplemental Figure 5

Figure S5. SSU peak coverage in control and 3-AT treated cells. 'SSU peak coverage' of mRNAs was defined as follows for each gene: First, the highest peak of footprints (FPs) in the 5' UTR was identified and measured; second, the height of the main-AUG peak was measured; finally, the peak coverage was calculated by dividing the 5' UTR FPs by the sum of the 5' UTR and main-AUG FPs. Data from 3 replicates was merged prior to plotting. **A-B:** Untreated cells. Both panels show the same plot, upper panel shows ribosomal genes selected in cyan. **C-D**: As A-B but cells treated with 3-AT. Genes discussed in the main text are highlighted, ribosomal gene on upper panels, non-ribosomal genes on lower panels.

Overlap of 3-AT regulated transcripts
between Ribo-Seq and TCP-seq

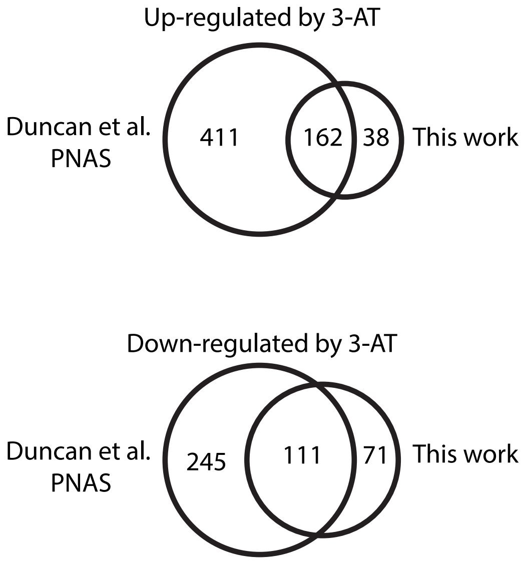

Duncan & Mata (2022), Supplemental Figure 6

Figure S6. Comparison of RNA-seq results from TCP-seq and standard RNA-seq libraries. Venn diagrams comparing differentially expressed genes upon 3-AT treatment from Duncan et al. PNAS 2019 (standard RNA-seq) and those identified in this work.

Relative FPs (5′ or 3′ ends mapped) of 40S and 80S libraries for untreated vs 3-AT treated cells

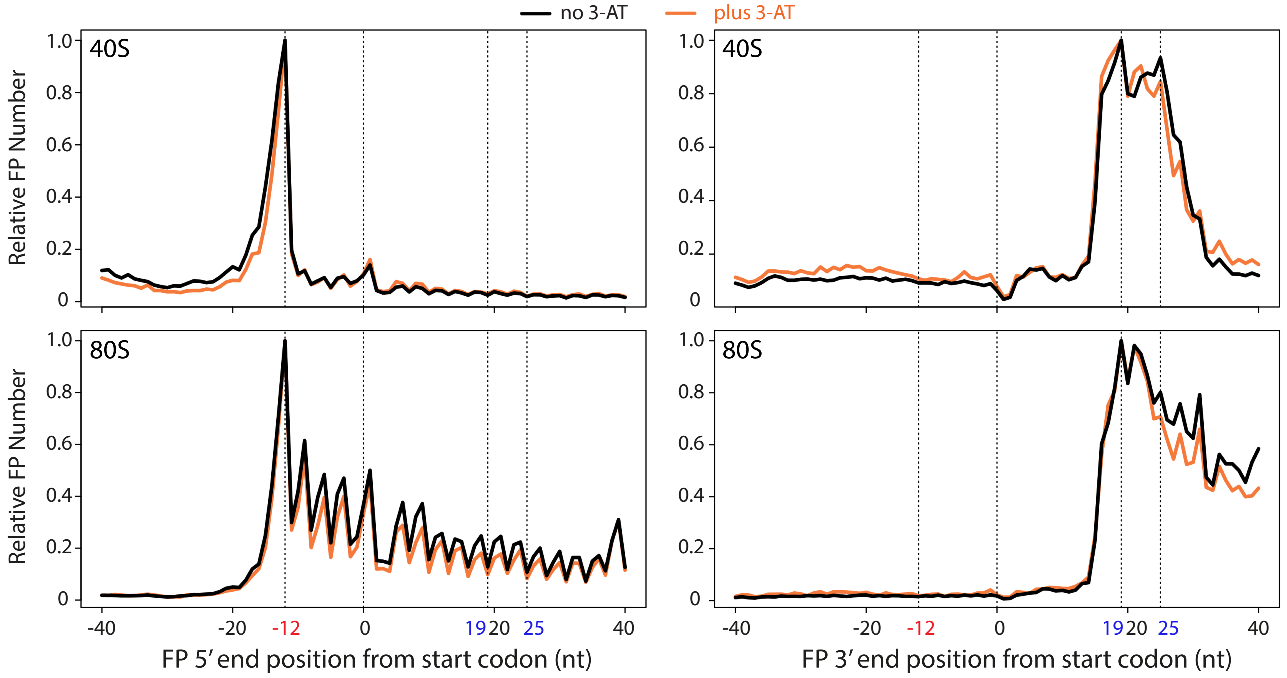

Duncan & Mata (2022), Supplemental Figure 7

Figure S7. Distribution of footprints (FPs) around translation initiation sites upon 3-AT-treatment. The distance of the FPs to the initiation site is displayed for the 5′ end (left) and the 3′ end (right). Top (40S) and bottom (80S). Data are shown for control cells (black) and 3-AT-treated cells (orange). The plots are similar to those on Figures 1D, 1E and 2C, 2D but also include 3-AT-treated cells. Translation start site is indicated with a dotted line labelled 0. Dotted lines at -12, 19, and 25 highlight peaks of interest.

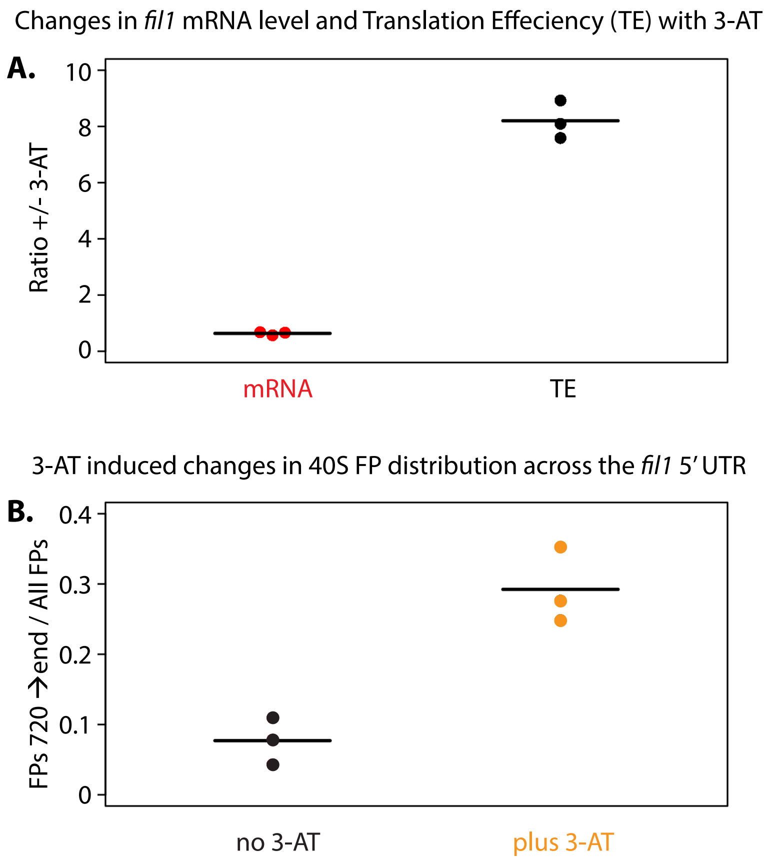

Duncan & Mata (2022), Supplemental Figure 8

Figure S8. Regulation of the *fil1* gene by 3-AT treatment. A. Changes in mRNA levels (left) or translational efficiency (right) upon 3-AT treatment (ratio +/- 3-AT). **B.** Changes in the distribution of 40S FPs on *fil1* 5' UTR upon 3-AT treatment. The plot shows the number of FPs in nucleotides 720-1200 of the 5' UTR, normalised by the total number of FPs, in control cells (left) and 3-AT treated cells.

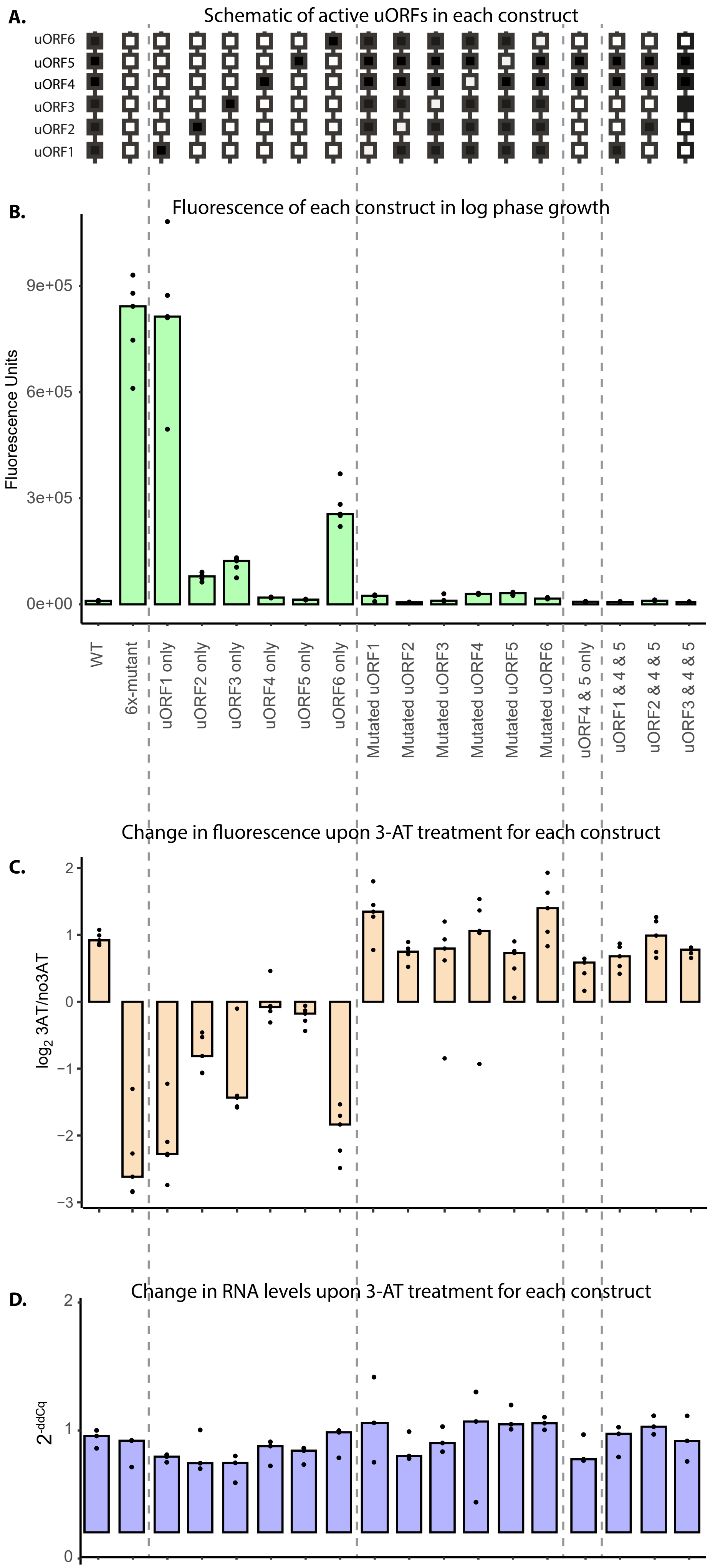

Duncan & Mata (2022), Supplemental Figure 9

Figure S9. Reporter analysis of the *fil1* 5' UTR. A. Schematic of reporter constructs generated and analysed in this work. Black squares indicate a functional uORF, whereas white squares represent uORFs inactivated by mutation. **B.** Fluorescence levels of reporters containing the indicated versions of the *fil1* 5' UTR in the absence of 3-AT (arbitrary units). **C.** Changes in fluorescence reporter levels expressed as the ratio between 3-AT-treated and untreated control cells (log_2-transformed). Data are from 5 independent biological replicates (dots represent the values from each experiment and bars show the corresponding means). **D.** Changes in mRNA reporter levels measured with RT-qPCR between 3-AT-treated and untreated control cells. Dots represent 3 biological repeats, with bars indicating the means. Calculations were performed as described in Materials and Methods.
